# Psilocybin in Older Adults: Therapeutic Opportunities in Inflammation-Driven Disorders of Aging—From Depression to Neurodegeneration

**DOI:** 10.3390/ijms27104229

**Published:** 2026-05-09

**Authors:** Marta Jóźwiak-Bębenista, Anna Stasiak, Monika Sienkiewicz, Paweł Kwiatkowski, Edward Kowalczyk

**Affiliations:** 1 Department of Pharmacology and Toxicology, Medical University of Lodz, Zeligowskiego 7/9, 90-752 Lodz, Poland; edward.kowalczyk@umed.lodz.pl; 2 Department of Hormone Biochemistry, Medical University of Lodz, Zeligowskiego 7/9, 90-752 Lodz, Poland; anna.stasiak@umed.lodz.pl; 3 Department of Pharmaceutical Microbiology and Microbiological Diagnostic, Medical University of Lodz, Muszynskiego 1, 90-151 Lodz, Poland; monika.sienkiewicz@umed.lodz.pl; 4 Department of Diagnostic Immunology, Pomeranian Medical University in Szczecin, Powstańców Wielkopolskich 72, 70-111 Szczecin, Poland; pawel.kwiatkowski@pum.edu.pl

**Keywords:** psilocybin, inflammation, aged, 5-HT_2A_, receptor, serotonin, neuronal plasticity, depression, neurodegenerative diseases

## Abstract

Aging is associated with chronic, low-grade inflammation (“inflammaging”), which contributes to neuropsychiatric and neurodegenerative disorders such as depression, Alzheimer’s disease, and Parkinson’s disease. Conventional pharmacotherapies often provide limited benefit in older adults and are further complicated by polypharmacy and drug–drug interactions. Psilocybin, a serotonergic psychedelic acting primarily as a partial agonist at the 5-HT_2A_ receptor and currently undergoing accelerated clinical development, has emerged as a potential multimodal therapeutic agent addressing these challenges. Acting via its active metabolite psilocin, 5-HT_2A_ receptor-mediated signaling modulates cortical glutamatergic transmission, enhances tropomyosin receptor kinase B/brain-derived neurotrophic factor (TrkB/BDNF) pathways, and modulates neuroimmune cascades (includingnuclear factor kappa B (NF-κB), with convergent systems-level effects such as reorganization of the default mode network. Human studies report acute reductions in TNF-α with variable effects on IL-6 and CRP, consistent with an immunomodulatory profile. Pharmacokinetically, psilocybin shows properties advantageous in geriatric care: rapid onset, short half-life, and predominant phase-II glucuronidation, reducing interaction risk. Controlled studies demonstrate rapid antidepressant and anxiolytic effects in major depressive disorder, treatment-resistant depression, and existential distress, with emerging feasibility signals in neurodegeneration. Together, these findings support the hypothesis that a time-limited, mechanism-based intervention may improve mood and cognition while attenuating inflammation. This review integrates current evidence on psilocybin’s neuroimmune and pharmacokinetic mechanisms relevant to aging, outlining its potential role in inflammation-related disorders and highlighting the need for targeted studies in older adults, who remain underrepresented in psychedelic research.

## 1. Introduction

According to the World Health Organization (WHO), the proportion of the global population aged 65 and over is projected to nearly double, from 12% in 2015 to 22% by 2050. More than 20% of older adults suffer from either a mental or neurological disorder, with depression and dementia being among the most prevalent neuropsychiatric conditions in this age group [1,2].

Aging is increasingly recognized as a state of chronic, low-grade systemic inflammation—a phenomenon often referred to as inflammaging—characterized by elevated levels of pro-inflammatory cytokines such as interleukin-6 (IL-6), tumor necrosis factor-alpha (TNF-α), and C-reactive protein (CRP) in the absence of active infection. This immunosenescent state contributes to the pathogenesis of numerous age-related conditions, including cardiovascular disease, sarcopenia, frailty, and neuropsychiatric disorders [3,4,5].

Neuroinflammation, in particular, plays a central role in the progression of various neurodegenerative and psychiatric disorders, including Alzheimer’s disease (AD), Parkinson’s disease (PD), multiple sclerosis (MS), amyotrophic lateral sclerosis (ALS), and major depressive disorder (MDD) [6,7]. These conditions are characterised by chronic microglial activation, blood–brain barrier (BBB) disruption, oxidative stress, and dysregulated cytokine production, which together contribute to neuronal injury, demyelination, and synaptic dysfunction [8,9].

In AD, pro-inflammatory cytokines and microglial activation promote amyloid-β aggregation and tau hyperphosphorylation, thereby exacerbating cognitive decline [10]. In PD, neuroinflammation contributes to degeneration of dopaminergic neurons in the substantia nigra [11]. In MS, immune-mediated demyelination and glial activation lead to lesion formation and progressive disability [12], whereas in ALS, inflammatory responses correlate with motor neuron loss and disease progression [13]. Similarly, after traumatic brain injury (TBI) or stroke, neuroinflammation sustains secondary neuronal damage and impairs long-term recovery [14,15].

Moreover, elevated concentrations and activity of immune cells, along with increased levels of pro-inflammatory cytokines, have been observed in a subset of patients with depression, especially those with treatment-resistant depression (TRD) and suicidal behaviour [16,17]. Depression itself has been associated with an increased risk of subsequent dementia, suggesting a bidirectional and biologically intertwined relationship between affective and neurodegenerative pathology [18]. In older adults, this inflammatory burden is further exacerbated by elevated levels of pro-inflammatory cytokines (IL-6, TNF-α, IL-1β), dysregulation of the hypothalamic–pituitary–adrenal (HPA) axis, and disturbances in the gut microbiota [19].

Given the limitations of conventional therapies, which have shown limited long-term efficacy in managing chronic neuroinflammation, especially in older populations, there is an urgent need for novel interventions that can precisely modulate inflammatory pathways while simultaneously improving mood, cognition, and functional status.

Rapid-acting agents with antidepressant properties, including serotonergic psychedelics such as psilocybin, have attracted attention as candidate multimodal therapeutics. In clinical studies, psilocybin has shown promise for major depressive disorder (MDD), TRD, anxiety, and existential distress associated with life-threatening illness, with benefits that can persist for months after a single administration [20,21]. These compounds exert multimodal effects by modulating neuroplasticity, serotonergic and glutamatergic signaling, and immune activity [22]. Psychedelics have demonstrated the ability to reduce pro-inflammatory cytokine expression, regulate microglial activation, and promote synaptogenesis, making them attractive therapeutic tools for inflammation-driven psychiatric and neurodegenerative conditions [9,23,24].

Because inflammaging and multimorbidity frequently co-occur in older adults, the rationale for interventions that target both the immune and neural systems simultaneously is compelling. Polypharmacy, prevalent in this population, is associated with increased risks of cognitive impairment, drug–drug interactions, and poor adherence [25]. In geriatric care, the priority is often to simplify pharmacological regimens rather than to add new medications. Therefore, therapeutic agents that offer multimodal benefits—anti-inflammatory, neuroplastic, and mood-stabilizing effects—in a single compound are of particular interest in this population.

In this narrative review, we focus on psilocybin, a serotonergic psychedelic whose active metabolite, psilocin, acts primarily as a partial agonist at the 5-HT_2A_ receptor and has the most advanced clinical evidence among psychedelic compounds. As the field of psychedelic-assisted therapy evolves, it is essential to critically assess existing data on its efficacy, safety, and underlying mechanisms, particularly in older adults, who are often burdened by complex comorbidities such as depression, anxiety, existential distress, chronic pain, and neurodegenerative conditions.

**Figure 1:**
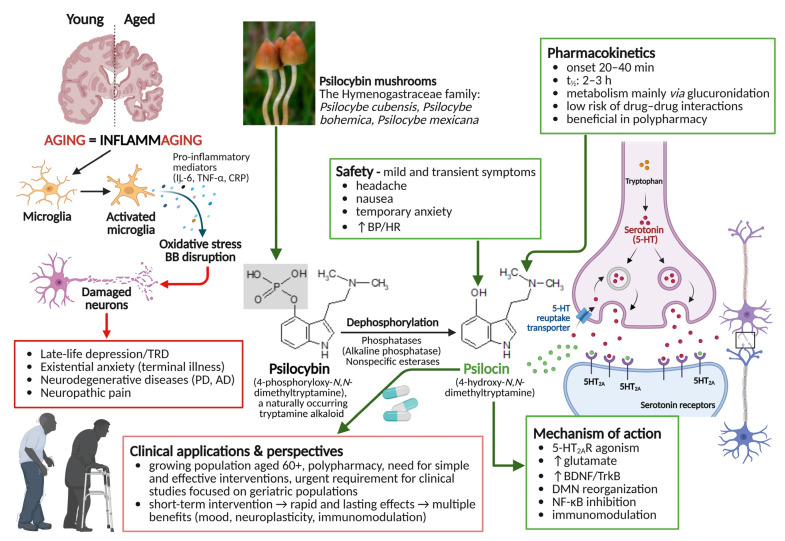
Overview of psilocybin in the elderly: from natural origin to synaptic action. Green arrows/text indicate beneficial or therapeutic effects/pathways, whereas red arrows/text indicate pathological or inflammation-related processes. Created in BioRender, Stasiak, A. (2026) https://BioRender.com/6h2q8mg (licensed under CC BY 4.0) and MDL ISIS Draw version 2.3 (chemical formulas).

The aim of this review is to evaluate the therapeutic potential of psilocybin in geriatric neuropsychiatry by examining its pharmacokinetics, mechanisms of action, clinical efficacy, and safety, with a focus on inflammation-related disorders of aging. More importantly, we seek to highlight the critical need for greater inclusion of older adults in clinical trials involving psychedelics—a group that remains severely under-represented despite potentially benefiting the most from such interventions (Figure 1).

## 2. Psilocybin

Psilocybin has emerged as a compound of growing scientific interest for its unique ability to modulate consciousness, cognition, and emotional states. It is a naturally occurring tryptamine-derived psychedelic found in certain species of mushrooms, such as *Psilocybe cubensis* and *Psilocybe Mexicana*. These so-called “magic mushrooms” have a long history of ritual and therapeutic use among indigenous cultures in Central and South America. Archaeological findings, including ceremonial “mushroom stones,” suggest that psilocybin-containing mushrooms were used as early as 3000 years ago. The Aztecs called them *teonanacatl*, “the flesh of the gods,” and regarded them as sacred tools for spiritual insight and healing [26].

Modern scientific interest in psilocybin began in the late 1950s, when Swiss chemist Albert Hofmann first isolated and synthesised the compound. In the 1960s, psilocybin attracted broader attention in psychiatric research, notably through studies by Walter Pahnke and Timothy Leary that explored its effects on consciousness, behaviour, and mental health [27].

However, widespread recreational use and the era’s countercultural movement led to growing concerns about public safety and misuse. As a result, in 1963, the United States imposed strict legal restrictions on the production, distribution, and use of serotonergic psychedelics, including psilocybin and lysergic acid diethylamide (LSD). Psilocybin was subsequently classified as a Schedule I substance under the Controlled Substances Act, defined as having a high potential for abuse and no accepted medical use. Similar restrictions were adopted in many other countries, effectively halting research for decades [28].

In recent years, growing interest in psychedelic-assisted therapies has renewed attention to psilocybin’s clinical potential. Several preclinical and clinical studies have demonstrated its potential efficacy in treating a range of psychiatric conditions, including depression, anxiety, and substance use disorders. In recognition of these findings, the U.S. Food and Drug Administration (FDA) granted psilocybin “Breakthrough Therapy” designation in 2018 for its use in TRD, thereby facilitating accelerated clinical evaluation and regulatory support [29].

While regulatory progress has been most notable in the United States, European frameworks have been slower to adapt [30]. Given the rising global burden of mental illness and the specific needs of aging populations, including military veterans exposed to chronic psychological stress, there is growing momentum to accelerate clinical research and policy reform. Recent geopolitical events, including large-scale armed conflicts, may further catalyse interest in novel therapies to address trauma, depression, and existential distress in affected populations.

## 3. Pharmacokinetics of Psilocybin

Understanding the pharmacokinetic profile of psilocybin is essential for interpreting its therapeutic effects, safety profile, and clinical applicability, particularly in older adults exposed to polypharmacy and age-related physiological changes. Psilocybin is a prodrug whose pleiotropic effects are mediated by its pharmacologically active metabolite, psilocin, which is formed rapidly during first-pass metabolism via dephosphorylation by alkaline phosphatases and non-specific esterases. Following oral administration, plasma psilocin reaches peak concentrations at approximately 2 h (C_max_ 15–20 ng/mL), whereas subjective effects begin within 20–40 min after dosing, peak at 60–90 min, and generally persist for 4–6 h. The elimination half-life of psilocin is approximately 2–3 h. These pharmacokinetic parameters have been reported in studies using both fixed oral dosing (e.g., 25 mg) and weight-adjusted regimens (typically 0.2–0.3 mg/kg) [31,32,33].

**Figure 2:**
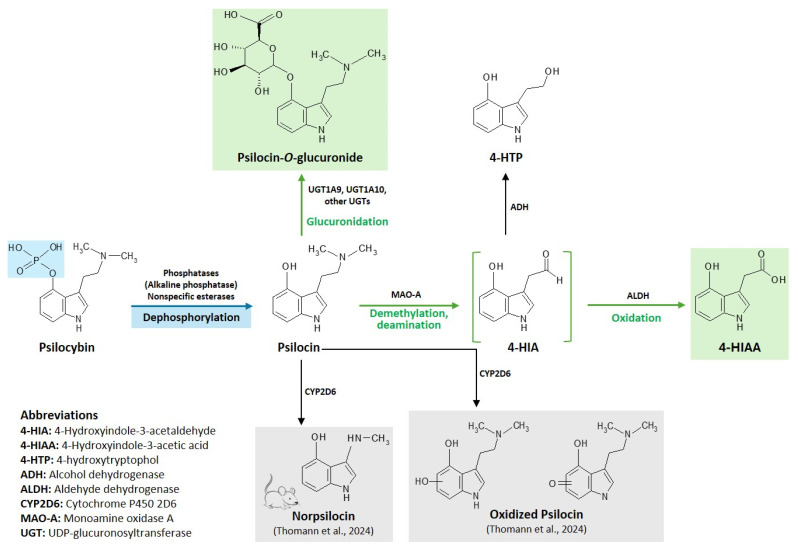
Synthesis and metabolic pathways of psilocin. Created with MDL ISIS Draw version 2.3. The primary active metabolites (highlighted in green) include psilocin-O-glucuronide (the most abundant) and 4-Hydroxyindole-3-acetic acid (4-HIAA). The intermediate metabolite (4-HIA, 4-Hydroxyindole-3-acetaldehyde) is shown in green brackets, while potential or unconfirmed metabolites are displayed on a grey background [35].

Approximately 80% of psilocin undergoes phase II metabolism, predominantly via glucuronidation catalyzed by UGT1A10 (intestinal) and UGT1A9 (hepatic) isoenzymes (Figure 2). The resulting metabolite, psilocin-O-glucuronide, is pharmacologically inactive and is excreted primarily in urine, with elimination largely complete within 24 h. Only approximately 1.5% of the administered psilocybin dose is excreted unchanged as free psilocin [34].

The remaining fraction of psilocin is metabolized via minor oxidative pathways involving monoamine oxidase (MAO) and aldehyde dehydrogenase (ALDH), yielding 4-hydroxyindole-3-acetaldehyde (4-HIA) as an intermediate. This compound is then converted either to 4-hydroxyindole-3-acetic acid (4-HIAA), a major urinary metabolite, or to 4-hydroxytryptophol (4-HTP), likely via alcohol dehydrogenase (ADH) [36].

From a clinical perspective, this metabolic profile may confer several potentially relevant advantages in later life. The predominant reliance on phase II conjugation, which is generally better preserved with aging than phase I oxidative metabolism, together with minimal CYP450 involvement, suggests a comparatively lower potential for pharmacokinetic drug–drug interactions than many conventional psychotropics or certain other serotonergic compounds [33]. This consideration is particularly relevant for patients with multimorbidity and polypharmacy. Across the studied dose ranges, psilocin exhibits approximately linear pharmacokinetics, and interindividual variability does not appear to be meaningfully explained by body weight [37]. Additionally, the relatively short half-life of psilocin (2–3 h) may reduce the risk of drug accumulation and allow for time-limited therapeutic windows. Compared with commonly prescribed psychotropic agents—such as benzodiazepines, which often have prolonged half-lives and are associated with sedation, falls, and cognitive impairment, or antipsychotics, which may confer extrapyramidal and metabolic adverse effects—the pharmacokinetic and administration profile of psilocybin may offer practical and safety-related advantages, particularly in older adults when used within structured clinical settings.

## 4. Mechanism of Action of Psilocybin

The therapeutic effects of psilocybin are mediated by psilocin, its pharmacologically active metabolite. Psilocin is a tryptamine structurally analogous to serotonin (5-HT) and acts primarily as a partial agonist at the 5-HT_2A_ receptor subtype. Earlier models proposed that psychedelic ligands exhibit functional selectivity (biased agonism), preferentially engaging Gq/PLC-linked signalling pathways rather than recruiting β-arrestin. However, more recent in vitro evidence suggests that classical psychedelics may act as partial, relatively unbiased agonists, with their distinct profile potentially reflecting greater signalling efficacy rather than pathway selectivity alone [38,39]. These receptors are highly expressed in brain regions involved in cognition, emotion regulation, and perception, including the prefrontal cortex, anterior cingulate cortex, and visual cortex [26,40].

The psychedelic effects of psilocin, understood as alterations in perception, cognition, and self-referential processing, are largely mediated by 5-HT_2A_ receptor activation and by downstream intracellular signalling patterns distinct from those induced by endogenous serotonin. This is associated with increased cortical glutamatergic signalling and subsequent activation of neuroplasticity-related cascades, including upregulation of brain-derived neurotrophic factor (BDNF) and engagement of tropomyosin receptor kinase B (TrkB) signalling [41,42]. Notably, recent data suggest that psilocin may also act as an allosteric modulator of TrkB, thereby enhancing BDNF-driven neuroplasticity independently of serotonergic pathways [43].

These mechanisms are particularly relevant in the context of aging, as neuroplasticity and BDNF signalling decline with age and are further impaired in neuropsychiatric and neurodegenerative disorders [44]. By modulating plasticity-related signalling, psilocybin may help counteract age-related synaptic rigidity and support adaptive emotional and cognitive functioning among older adults [39].

Psilocybin also modulates large-scale brain networks. Functional neuroimaging studies have consistently shown altered activity and connectivity within the default mode network (DMN)—a system implicated in self-referential thought, rumination, and affect regulation—which is often hyperactive in depression and functionally disrupted in aging and dementia [45,46,47]. In older adults, age-related changes in DMN integrity may influence both the subjective and therapeutic effects of psilocybin, possibly shifting the mechanism of benefit from intense perceptual experiences towards improved emotional regulation and psychological flexibility [48].

At the cellular level, preclinical studies suggest that psilocybin induces rapid structural plasticity, including increased dendritic spine density and synaptogenesis in key regions such as the medial prefrontal cortex and hippocampus, areas known to atrophy with aging and neuroinflammation. These neuroadaptive effects may underpin the sustained improvements in mood and anxiety observed even after a single administration, offering a potential therapeutic avenue for older adults with depression or neurodegenerative conditions [5,49].

Although 5-HT_2A_ receptor activation is central to psilocybin’s mechanism of action, psilocin also binds to other serotonergic receptor subtypes (5-HT_1A_, 5-HT_2C_, 5-HT_1D_) with lower affinity and modulates glutamatergic and dopaminergic systems [26]. Consistent with this, indirect dopaminergic modulation, including increased extracellular dopamine in frontal and limbic regions, has been reported following psilocybin administration, potentially enhancing reward processing and motivational drive, both of which may be blunted in late-life depression [50,51,52].

In sum, psilocybin engages a network of molecular and systems-level mechanisms—serotonergic signalling, glutamate transmission, neurotrophic modulation, and DMN reorganization—that may be particularly suited to addressing the multifactorial neurobiology of mood and cognitive disorders in older adults. Its ability to influence synaptic remodeling, emotional processing, and inflammation-related pathways distinguishes it from conventional monoaminergic antidepressants and highlights its potential for geriatric neuropsychiatry (Table 1).

## 5. Anti-Inflammatory Action of Psilocybin

Psilocybin has emerged as a compound with immunomodulatory properties, which may be particularly relevant in the context of inflammation-driven disorders of aging (Table 2). These effects are primarily mediated by agonism of the serotonin 5-HT_2A_ receptor, which is expressed in the central nervous system and on peripheral immune cells. Activation of these receptors has been implicated in regulating key inflammatory pathways, including modulation of pro-inflammatory cytokine production and NF-κB-dependent transcription [23,63].

Consistent with this framework, preclinical studies indicate that psilocybin and its active metabolite, psilocin, modulate immune responses in a context- and dose-dependent manner. In microglial models, psilocin attenuates inflammatory activation, reducing TLR4 expression and NF-κB signaling [64]. In macrophage systems, psilocin decreases TNF-α and increases IL-10, whereas psilocybin itself exhibits variable and context-dependent effects, influenced by the timing of administration and inflammatory state [65]. Importantly, recent in vitro data indicate that psilocybin does not exert uniformly anti-inflammatory effects but instead induces dose-dependent, bidirectional immune responses, including suppression of IL-1β, IL-6, and COX-2 at higher concentrations and pro-inflammatory signaling at lower doses [66,67]. Additional evidence from human-derived tissue models indicates that psilocybin may attenuate inflammatory cytokine responses, including TNF-α, IL-6, and MCP-1, under inflammatory stimulation; however, these findings are limited by supraphysiological concentrations and non-neuronal experimental systems [66]. In vivo, psilocybin has been reported to reduce markers of neuroinflammation in LPS-induced models, supporting its potential relevance to central inflammatory processes, although the effects are modest and context-dependent [58].

In humans, available evidence remains limited and inconsistent. In healthy volunteers, a single dose of psilocybin was associated with acute reductions in TNF-α and sustained decreases in IL-6 and C-reactive protein (CRP) [60]. In contrast, another study did not observe significant changes in inflammatory biomarkers, suggesting that these effects may depend on baseline inflammatory status, dosing, and the timing of assessment [59].

At the molecular level, 5-HT_2A_ receptor activation triggers intracellular signaling cascades via Gq/PLC pathways and downstream effectors such as MAPK/ERK, PI3K/Akt, and mTOR, which are implicated in both neuroplasticity and immune regulation [9]. Experimental studies using selective 5-HT_2A_ agonists support a mechanistic link between serotonergic signaling and inhibition of TNF-α and NF-κB pathways [68]. Notably, psychedelics may not act as classical anti-inflammatory agents but rather as modulators of neuroimmune signaling, shifting immune responses in a state-dependent manner.

Psilocybin may also influence inflammatory processes through serotonergic regulation of metabolic pathways linked to neuroinflammation, although direct evidence remains limited. In particular, modulation of the kynurenine pathway has been proposed as a potential mechanism linking serotonergic signaling to neuroimmune regulation [9,61]. In parallel, psilocybin has been shown to modulate glutamatergic transmission in cortical circuits, which may indirectly reduce excitotoxicity—a process closely associated with neuroinflammation [39,62].

Taken together, current evidence indicates that psilocybin exerts complex, context-dependent immunomodulatory effects rather than uniform anti-inflammatory effects. These properties, together with their effects on neuroplasticity, may be particularly relevant to disorders of aging characterized by chronic, low-grade inflammation.

**Table 2:** The anti-inflammatory effects of psilocybin across preclinical and human studies.

Evidence Level	Model	Intervention	Main Findings	Relevance/Limitation	Ref.
**Preclinical ** **(in vitro)**	LPS-stimulated RAW264.7 macrophages	Water and ethanol extracts of *Psilocybe natalensis*	Dose-dependent reduction of NO, PGE2, and IL-1β; increased IL-10 (ethanol extract); no major cytotoxicity at tested doses	Suggests anti-inflammatory potential of psilocybin-containing mushroom extracts; effects cannot be attributed solely to psilocybin	[69]
**Preclinical** **(in vitro)**	LPS-stimulated human U937 macrophages	Hot-water extracts of psilocybin-containing mushrooms (25–50 µg/mL); pre-treatment prior to LPS stimulation	Reduced TNF-α and IL-1β; decreased IL-6 and COX-2; non-significant increase in IL-10; minimal effect on 15-LOX	Suggests anti-inflammatory potential of mushroom extracts; effects cannot be attributed specifically to psilocybin	[70]
**Preclinical ** **(in vitro)**	Primary mouse CD11b+ microglia (±LPS); neuron–microglia co-culture	Psilocin (100 µM; 12 h exposure), with or without prior LPS stimulation	Reduced TLR4, NF-κB p65, and CD80 expression; increased TREM2; attenuated microglial phagocytosis of healthy neurons	Supports direct immunomodulatory and potentially neuroprotective actions of psilocin; preclinical in vitro data at supraphysiological concentrations	[64]
**Preclinical** **(in vitro)**	LPS-stimulated human THP-1 macrophages	Psilocybin (5–15 µM); co-treatment with LPS	Dose-dependent, bi-phasic effects: reduced IL-1β, COX-2, and IL-6 with inhibition of NF-κB/TYK2–STAT signaling and NLRP3 activation at higher concentrations, and pro-inflammatory responses at lower doses	Demonstrates dose-dependent, context-dependent immunomodulation in human macrophages; in vitro model with limited translational relevance	[67]
**Preclinical ** **(in vitro)**	TNF-α/IFN-γ-stimulated human 3D EpiIntestinal tissue	Psilocybin (10–40 µM)	Reduced TNF-α, IFN-γ, IL-6, IL-8, MCP-1, and GM-CSF levels	Demonstrates anti-inflammatory effects in human-derived tissue; however, the non-neuronal model limits direct applicability to CNS neuroinflammation	[66]
**Preclinical** **(in vitro)**	LPS-stimulated RAW264.7 macrophages	Psilocybin and psilocin (6–56 ng/mL; pre- and post-treatment relative to LPS stimulation)	Psilocin reduced TNF-α and increased IL-10 in LPS-activated macrophages; psilocybin showed mixed, context-dependent effects including transient TNF-α increase	Supports immunomodulatory effects; highlights dose-, timing-, and metabolite-dependent responses; in vitro model limits translational relevance	[65]
**Preclinical** **(in vivo)**	LPS-induced neuroinflammation in mice	Psilocybin (0.88 mg/kg, oral gavage) ± eugenol (1:10–1:50); pre- and post-treatment	Reduced expression of IL-6, TNF-α, and COX-2, particularly in combination with eugenol; psilocybin alone showed modest and variable effects	Provides evidence for central anti-inflammatory effects; interpretation limited by combination treatment and inconsistent effects of psilocybin alone	[58]
**Human experimental**	Healthy adult volunteers	Single oral psilocybin administration (25 mg)	Acute reduction in TNF-α; sustained decreases in IL-6 and CRP for up to 7 days; associated with improved mood and reduced stress reactivity	Provides controlled human biomarker evidence of systemic anti-inflammatory effects; conducted in healthy participants rather than clinical populations	[60]
**Human experimental**	16 healthy adult volunteers (open-label study)	Single oral psilocybin dose (mean 0.22 mg/kg; ~17.4 mg)	No statistically significant changes in hsCRP, TNF, or suPAR at 24 h; numerically, hsCRP decreased by 32%	First human study assessing inflammatory biomarkers; no robust peripheral anti-inflammatory effect after a single dose in healthy low-inflammation participants; limited by small sample size and open-label design	[59]

## 6. Clinical Applications in Inflammation-Linked Conditions

### 6.1. Depression and Anxiety

Psilocybin has demonstrated clinically meaningful antidepressant and anxiolytic effects in randomised and open-label clinical studies in populations with MDD and TRD. Symptom reduction typically occurs within days and, when embedded within structured psychotherapeutic support, may persist for weeks to months.

MDD/TRD. In MDD, a randomized, waiting-list–controlled study with two supported psilocybin dosing sessions (20 mg/70 kg followed by 30 mg/70 kg; mean interval of ~1.6 weeks) reported a 71% response rate and a 54% remission rate at 4 weeks, with sustained benefit in a substantial subset at 12 months [71,72]. In a head-to-head phase-2 trial, two 25 mg psilocybin sessions (3 weeks apart) outperformed escitalopram on multiple secondary outcomes, including changes in the Beck Depression Inventory (BDI-1A), Hamilton Depression Rating Scale (HAM-D-17), Montgomery–Åsberg Depression Rating Scale (MADRS), State–Trait Anxiety Inventory (STAI), and measures of well-being and functional impairment, with higher response (70% vs. 48%) and remission (57% vs. 28%) rates at week 6 [73]. In TRD, open-label protocols administering psilocybin at 10 mg followed by 25 mg seven days later produced marked short-term improvements, with persistence in a subset at 3–6 months [74]. A larger phase 2b study found that a single 25 mg session produced the greatest short-term improvement at week 3, with between-group differences narrowing by week 12—useful context when setting expectations for durability after one dose [75]. Complementing SSRI-washout paradigms, an exploratory open-label study in which a single 25 mg psilocybin session was added to ongoing SSRI therapy achieved week 3 response and remission rates of 42% each, indicating that efficacy signals can emerge without suspending background antidepressants [76].

Beyond symptomatic improvement, attention has increasingly focused on the role of inflammation in depression. Treatment-resistant and late-life depression are frequently associated with elevated inflammatory markers, and evidence suggests that patients with higher baseline inflammation may derive greater benefit from immunomodulatory interventions [17]. Although psilocybin has been proposed to interact with neuroimmune pathways, clinical evidence supporting its anti-inflammatory effects in patient populations remains limited.

Serious illness and death anxiety. Two double-blind, crossover RCTs in patients with life-threatening cancer demonstrated rapid and sustained reductions in anxiety and depressive symptoms after a single moderate-to-high psilocybin dose, with approximately 60–80% of participants maintaining clinically significant improvements at 6 months [31,77]. Reductions in death anxiety and changes in perceptions of mortality were assessed using validated instruments, including the Death Transcendence Scale. Patients frequently reported profound shifts in their perspectives on life and death, and the intensity of “mystical-type” experiences statistically mediated clinical benefit—phenomena particularly relevant to existential distress in later life [31,32].

### 6.2. Neurodegenerative Disorders

Across neurodegenerative diseases, neuroinflammation, synaptic vulnerability, and impaired neuroplasticity converge to create a shared therapeutic target profile for serotonergic psychedelics. Mechanistically, 5-HT_2A_–biased signalling has been shown to attenuate NF-κB–dependent transcription and peripheral cytokine production, with complementary effects on microglial states and mTOR/BDNF-linked structural plasticity—an immune-neuroplastic “double hit” particularly relevant to diseases such as PD and AD [24,78]. Recent preclinical evidence further supports a potential role of psilocybin in modulating biological aging processes. In a murine model, psilocybin administration increased survival in aged animals and extended cellular lifespan, effects associated with reduced oxidative stress, preservation of telomere length, and upregulation of longevity-related pathways such as SIRT1 signalling [79].

Parkinson’s disease. First-in-disease clinical data now indicate that psilocybin therapy is feasible in PD and may benefit domains beyond mood. In an open-label pilot (NCT04932434), 12 adults with mild–moderate PD and comorbid depression and/or anxiety received two supported doses (10 mg then 25 mg) alongside structured psychotherapy. The intervention was well tolerated, with no serious adverse events or exacerbation of psychosis. Clinically, non-motor symptoms and activities of daily living improved substantially, with smaller but significant gains on standardized motor assessments and better performance on selected cognitive tasks in memory and executive function. Depressive and anxiety symptoms decreased and remained lower at 3 months in exploratory follow-up. Notably, nine participants continued on stable carbidopa–levodopa without apparent signs of interaction [80]. Complementing these pilot data, a detailed case report described a 43-year-old person with PD without depression who underwent four high-dose psilocybin-assisted psychotherapy sessions over one year. Treatment was well tolerated and associated with durable reductions in anxious ruminations, improved dispositional optimism and disease acceptance, and stable motor status, underscoring potential quality-of-life benefits even when a mood disorder is subthreshold [81]. Together, these signals justify controlled trials to clarify the specific effects on mood, non-motor burden, motor function, and cognition in PD.

Alzheimer’s disease. Initial clinical trials are underway: an open-label pilot at Johns Hopkins is testing two psilocybin sessions at weeks 4 and 6, embedded within eight weeks of weekly psychological support, to alleviate depressive symptoms in people with MCI or early AD, with outcomes tracked for up to ~6 months (NCT04123314).

Neuropathic pain. Early evidence suggests that psilocybin may provide clinically meaningful analgesia for neuropathic pain. Case reports and small series in conditions such as complex regional pain syndrome and phantom-limb pain describe marked reductions in pain intensity and interference, sometimes accompanied by reduced analgesic use, after one or a few supported dosing sessions [82,83]. Preclinical data align with these observations: a single psilocybin dose produces persistent antinociception in neuropathic models and engages 5-HT_2A_-linked plasticity mechanisms relevant to central sensitization [83,84]. Trial readiness also appears high—most patients (~77%) in the PEACE-PAIN prospective assessment expressed willingness to enrol in an RCT, citing the importance of credible controls and structured psychological support [85].

**Figure 3:**
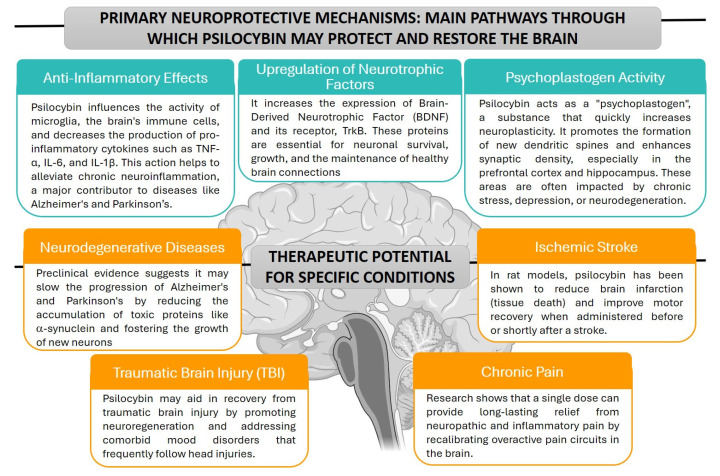
Main pathways through which psilocybin may protect and restore the brain and its therapeutic potential in CNS diseases. Brain icon was created in BioRender. Stasiak, A. (2026) https://BioRender.com/o7xxvb2 (licensed under CC BY 4.0).

Figure 3 summarizes the basic neuroprotective mechanisms of psilocybin and its therapeutic potential for treating CNS diseases.

### 6.3. Effectiveness of Psilocybin in Older Adults

#### 6.3.1. Neurobiological Considerations

Given psilocybin’s favourable pharmacokinetic profile in older adults—including preserved phase II metabolism, low risk of CYP450 interactions, and a short half-life—an important question arises: can psilocybin be equally effective in this population, whose neurobiology may differ significantly from that of younger patients? The effectiveness of psilocybin in this population may be modulated by age-related alterations in the serotonergic system. While serotonergic neuron firing rates and central serotonin synthesis decline with age, this may not necessarily impair the pharmacodynamic response to psilocybin [86]. Unlike selective serotonin reuptake inhibitors (SSRIs), psilocybin, via its active metabolite, acts as a direct partial agonist at 5-HT_2A_ receptors, thereby bypassing the need for elevated endogenous serotonin levels.

Moreover, recent meta-analytic data indicate that serotonin transporter (SERT) availability declines moderately with age, potentially prolonging synaptic serotonin activity by slowing reuptake [87]. This compensatory mechanism could mitigate the impact of reduced presynaptic activity and support sufficient receptor engagement. Importantly, vesicular monoamine transporter 2 (VMAT2)—which packages serotonin into presynaptic vesicles—appears to remain largely stable across the adult lifespan, suggesting that vesicular 5-HT content is preserved in the aging brain [88]. However, the density and functional sensitivity of 5-HT_2A_ receptors, which mediate the psychedelic effects of psilocybin, may also decline with age, particularly in cortical regions involved in mood and cognition [89]. As a result, older adults might exhibit greater variability in how they perceive responses, tolerate treatments, and determine optimal dosing.

In older adults, age-related gut dysbiosis and impaired intestinal barrier integrity may be clinically relevant modifiers of psilocybin response. Aging is associated with increased intestinal permeability, systemic translocation of microbe-derived products, and chronic low-grade inflammation, all of which may contribute to neuroinflammatory vulnerability [90]. Emerging experimental evidence further indicates that psilocybin may directly affect peripheral gut physiology. In a recent mouse study, chronic psilocybin administration produced dose-dependent changes in gut motility and altered specific microbial taxa, including *Lactobacillus* and *Alistipes* species, with sex-dependent effects observed predominantly in male animals [91]. In parallel, the concept of the “psilocybiome” suggests that baseline microbiome composition may influence preparation, acute treatment response, and post-session integration, thereby contributing to inter-individual variability in psychedelic outcomes [92]. Because orally administered psilocybin undergoes gastrointestinal absorption and is converted to psilocin, age-related changes in gut function and microbiome composition may partly influence tolerability, pharmacokinetic variability, and therapeutic response in later life.

Beyond age-related central and peripheral changes, biological sex may further modulate 5-HT_2A_ receptor signalling and responsiveness to psilocybin. Sex differences in serotonergic neurotransmission, cortical receptor expression, and hormone-dependent plasticity are well documented across the lifespan, suggesting that menopausal and postmenopausal endocrine changes may alter sensitivity to psilocin in older women [26,89]. Emerging preclinical evidence also indicates sex-specific responses to psychedelics at behavioral, circuit, and molecular levels. A recent preclinical study demonstrated that psilocybin reduced conditioned opioid reward in male but not female mice via 5-HT_2A_ receptor activation in frontal cortex neurons projecting to the nucleus accumbens, accompanied by sex-dependent enhancer activity and changes in structural plasticity [93]. More broadly, sex-dimorphic patterns of vulnerability and resilience to stress-related disorders have been reported in rodent models, including female-predominant depressive-like phenotypes associated with distinct signatures of hippocampal neuroplasticity and oxidative stress [94]. These observations suggest that sex-specific neurobiology may contribute to interindividual variability in psilocybin efficacy, tolerability, and optimal dosing in later life, underscoring the importance of sex-stratified analyses in future clinical trials with older adults.

Overall, whether these age-related modifiers result in diminished, preserved, or heightened sensitivity to psilocybin remains unclear and likely varies between individuals depending on receptor expression, gut physiology, inflammatory status, and cortical plasticity.

#### 6.3.2. Emerging Clinical Evidence

Although older adults remain severely underrepresented in clinical trials involving psychedelic compounds—with only 1.4% of participants aged 65 or older enrolled in such studies since 1965—a recent systematic review underscores the urgent need for age-inclusive research in this field [20]. In one of the earliest prospective observational studies focused specifically on this population, Kettner and colleagues (2024) followed 62 individuals aged 60 years or older who voluntarily participated in group-based psilocybin ceremonies [48]. The study found statistically significant improvements in psychological well-being, as measured by the Warwick-Edinburgh Mental Well-being Scale (WEMWBS), particularly at two and four weeks post-session. Notably, these effects were most pronounced among participants with a history of mental health diagnoses, suggesting potential relevance of psilocybin-assisted interventions for older adults with longstanding psychological distress.

Interestingly, although older adults reported lower intensity of classic psychedelic experiences—such as ego dissolution or mystical-type states—than younger controls, their improvements in well-being were comparable. This suggests that therapeutic efficacy in older adults may be mediated by partially distinct mechanisms. The attenuated acute phenomenology may reflect age-related reductions in cortical 5-HT_2A_ receptor density, together with altered responsiveness of large-scale brain networks, particularly the DMN, while still permitting clinically meaningful psychological benefit. Indeed, in this age group, the strongest predictor of improved outcomes was the degree of *communitas*—a sense of emotional connectedness and belonging experienced during the group session. These findings indicate that relational and contextual factors may be particularly important therapeutic mediators in later life, potentially compensating for attenuated acute psychedelic effects. However, interpretation of these findings requires caution, as the study used a naturalistic observational design, involved self-selected participants, and was conducted in group ceremonial settings, which may limit generalizability to routine clinical practice.

## 7. Adverse Effects and Safety Considerations of Psilocybin

Psilocybin was classified as a Schedule I substance, in part due to concerns about its potential to trigger persistent psychiatric conditions, such as psychosis [95]. However, subsequent analyses have shown that these risks are substantially elevated primarily among individuals with a personal or family history of psychotic disorders. For example, a recent case series examining the long-term psychological consequences of psychedelic use found that all participants who received a psychiatric diagnosis after a psychedelic experience had pre-existing mental illness [96].

Psilocybin-assisted therapy has consistently demonstrated a favourable safety profile when administered in controlled clinical settings with appropriate psychological support. Across multiple trials, most reported adverse events have been transient, mild to moderate in intensity, and typically confined to the acute drug-effect window [97,98]. The most commonly observed somatic symptoms include headache, nausea, and dizziness, which generally resolve spontaneously without medical intervention [98]. Transient psychological discomfort, such as anxiety or emotional unease, has also been noted but was rarely associated with lasting consequences when managed by trained therapists [74].

Importantly, extensive participant screening has played a critical role in minimizing the incidence of serious adverse events. Most clinical protocols excluded individuals with a personal or family history of psychosis or bipolar disorder—conditions considered relative contraindications to psychedelic therapy [71,74]. When such precautions are taken, the risk of severe psychiatric sequelae appears low in screened populations. A 12-month follow-up study found no evidence of persistent perceptual disturbances, emergent psychosis, or signs of dependence after psilocybin treatment [71].

A recent comprehensive safety review of 214 studies (including more than 3500 participants) reported delayed serious adverse events in 3.9% (23/584) of outpatients in high-dose psilocybin trials, predominantly among those with pre-existing neuropsychiatric disorders. These events included transient suicidality, worsening depression, and isolated episodes of psychosis or convulsions. Notably, no serious adverse events were observed in healthy individuals [97]. However, the analysis highlighted considerable heterogeneity in the monitoring and reporting of adverse events—only 23.5% of studies used systematic pharmacovigilance protocols—indicating a need for greater consistency in future research.

Although generally well tolerated, psilocybin may cause transient increases in blood pressure and heart rate, which could pose a risk for older adults with poorly controlled cardiovascular disease [99] (Table 3). Nevertheless, compared with conventional psychotropic agents commonly prescribed for geriatric populations—such as benzodiazepines or antipsychotics—psilocybin may pose a lower risk of cognitive impairment, sedation, falls, and extrapyramidal symptoms [73]. Given the high prevalence of polypharmacy in later life, potential drug–drug interactions should also be considered. Psilocin is metabolized predominantly via phase II glucuronidation, with limited CYP450 involvement, which may reduce the likelihood of multiple metabolic drug–drug interactions compared with many psychotropic and cardiovascular medications commonly used in older adults. However, pharmacodynamic considerations remain relevant, particularly in patients receiving antihypertensive, serotonergic, or CNS-active medications. Because psilocybin is typically administered intermittently rather than chronically, the cumulative interaction burden may be lower than with chronically administered daily therapies. Frailty, reduced physiological reserve, and multimorbidity may further increase vulnerability to acute haemodynamic or psychological stressors during psilocybin administration.

While the overall safety profile of psilocybin is reassuring, data on adverse effects in older adults remain sparse. In a recent systematic review, detailed safety data were available for only 10 older adults (≥60 years) who received psilocybin-assisted therapy for serious illness [20]. Among them, four individuals reported mild-to-moderate, transient psychiatric adverse events—primarily anxiety, paranoid ideation, or thought disturbance—all of which were successfully managed by study therapists without the need for pharmacological intervention or psychiatric hospitalization. No cases of persistent psychosis or hallucinogen-persisting perceptual disorder were observed. Transient episodes of hypertension were observed during dosing, with the highest recorded systolic pressure at 186 mmHg. These did not require medical intervention or result in long-term sequelae. Such haemodynamic responses may be clinically relevant in older individuals with underlying cardiovascular disease. Additionally, time-limited headaches and mild gastrointestinal symptoms were reported in a small subset [77,101]. These findings suggest that psilocybin, when administered in controlled clinical settings, may be well tolerated even among older adults. However, the small sample sizes and the absence of systematic age-stratified reporting across most trials highlight the urgent need for dedicated safety studies in geriatric populations.

In contrast to controlled clinical settings, a recent case report described prolonged and clinically significant adverse effects in a 71-year-old participant following repeated high-dose psilocybin use in an unregulated training environment, ultimately requiring electroconvulsive therapy [102]. Although this case does not reflect the safety profile observed in regulated clinical trials, it underscores the importance of careful screening, dose control, and structured clinical oversight, particularly for older adults.

## 8. Perspectives

As the global population ages, the prevalence of chronic neuroinflammatory and neuropsychiatric disorders—including treatment-resistant depression, anxiety, neurodegeneration, and multimorbidity—continues to rise. Older adults often require multiple medications to manage these conditions, increasing the risk of polypharmacy, adverse drug interactions, and treatment non-adherence. Psychedelic-assisted therapy, particularly with psilocybin, offers the possibility of addressing multiple age-related pathologies through a single pharmacological intervention that modulates both immune and neural pathways [25].

Given its anti-inflammatory, neuroplastic, and affective-regulatory properties, psilocybin may serve as a multimodal therapeutic agent for this population. However, this potential can only be realised through rigorous clinical investigation that includes older adults, who have thus far been routinely excluded from psychedelic trials. Importantly, this gap is now being addressed by large-scale initiatives such as the NIH-funded INSPIRE Network (1UG3AG094957–01), which aims to establish a geriatric psychedelic research consortium to evaluate the safety of psilocybin and LSD in older adults, followed by clinical trials targeting pain-related conditions. Carefully designed studies must account for the unique physiological and psychological characteristics of aging, including altered serotonin signalling, cardiovascular vulnerability, and emotional processing in later life. Equally important is ensuring that participants are cognitively intact and able to comprehend and integrate the psychedelic experience [9].

In parallel, regulatory reconsideration of psilocybin’s legal status may be warranted. In many jurisdictions, psilocybin remains in the most restrictive schedule (e.g., Schedule I), a classification that materially hinders clinical trials and the regulated supply. Reclassification from prohibition-only schedules to research-permissive categories (e.g., Schedule II/III, depending on jurisdiction) would lower administrative barriers, enable standardized manufacturing and controlled access for studies, and allow an evidence-based evaluation of benefit–risk while maintaining strict safeguards.

Despite growing enthusiasm, integrating psychedelics into mainstream medicine faces ethical, logistical, and societal challenges. Public perception remains clouded by decades of stigma linked to recreational use, and concerns persist over variability in response, the risk of abuse, and long-term safety [103]. Ethical concerns in clinical research are particularly pronounced in psychedelic therapy: informed consent must be robust, protocols must be trauma-informed, and long-term psychological support should be guaranteed [104,105]. The success of clinical interventions depends heavily on access to trained psychedelic therapists—currently a scarce resource—and on the infrastructure required to deliver preparation and integration, which can be resource-intensive and cost-prohibitive.

Addressing these barriers will require multi-level efforts, including standardized training programmes, development of clinical guidelines, public education, and progressive regulatory reform. Moreover, interdisciplinary research integrating neuroimaging, immunology, aging science, and psychotherapy is needed to optimize treatment protocols and ensure safety in vulnerable populations, such as older adults. The therapeutic promise of psilocybin is real, but its realization depends on the integrity, inclusivity, and sustainability of the systems we build around it.

## 9. Conclusions

Psilocybin is a mechanistically distinct therapeutic candidate for late-life neuropsychiatric disorders, particularly where depression, anxiety, chronic inflammation, and multimorbidity coexist. Its rapid central effects, downstream effects on neuroplasticity and immune-related pathways, short half-life, and limited CYP450 involvement may be advantageous in polypharmacy-prone older populations.

However, current enthusiasm outstrips the available age-specific evidence. Older adults remain markedly under-represented in psychedelic trials, and important uncertainties persist regarding optimal dosing, cardiovascular safety, cognitive vulnerability, sex-specific responses, and peripheral mechanisms, such as the microbiota–gut–brain axis. Emerging data further suggest that clinical benefit in later life may occur even when acute psychedelic experiences are attenuated.

Priority research needs include: (1) dedicated randomised controlled trials in adults aged ≥65 years; (2) pharmacokinetic and pharmacodynamic studies across strata of frailty and multimorbidity; (3) sex-stratified analyses; (4) long-term safety registries; and (5) biomarker-guided studies integrating neuroimaging, inflammatory markers, and functional outcomes.

At present, psilocybin should be regarded as an investigational intervention in geriatric neuropsychiatry. With rigorous regulation, therapist training, and age-adapted protocols, it may become a valuable option for selected older patients with limited therapeutic alternatives.

## Figures and Tables

**Table 1:** Mechanisms of action of psilocybin/psilocin and their relevance to aging. Summary of receptor-level, synaptic, intracellular, neuroimmune, and network-level processes potentially engaged by psilocybin/psilocin, with concise aging relevance statements (right column). ↑ indicates increase/upregulation; ↓ indicates decrease/downregulation.

Level	Mechanism/Target	Core Effect	Aging Relevance	Refs.
**Receptor**	Partial, biased agonism at 5-HT_2A_ (plus lower-affinity 5-HT_1A_/5-HT_2C_/5-HT_1D_)	Distinct intracellular signaling vs. endogenous 5-HT; drives psychedelic state	Preserved post-synaptic 5-HT_2A_ signaling may bypass age-related declines in presynaptic 5-HT tone	[26,40]
**Synaptic signaling**	5-HT_2A_-associated cortical glutamate increase; AMPA receptor recruitment	Triggers plasticity cascades (activity-dependent)	Counters age-related synaptic rigidity	[41,42]
**Neurotrophic ** **pathways**	BDNF ↑; TrkB engagement (allosteric modulation by psilocin)	Enhances synaptogenesis and spine formation	May restore plasticity pathways that decline with age	[43,44]
**Intracellular ** **cascades**	MAPK/ERK-, PI3K/Akt- and mTOR-related signaling	Gene programs supporting growth, survival and remodeling	Supports repair/adaptation in vulnerable aging circuits	[39,53,54]
**Network-level**	Default Mode Network (DMN) activity/connectivity modulation; network reorganization	Reduces rumination/self-referential rigidity; increases cognitive flexibility	DMN is altered in aging/dementia; modulation may aid mood/cognition	[45,46,47]
**Limbic processing**	Amygdala reactivity modulation	May improve processing of emotional salience	Targets affective dysregulation common in late-life depression/anxiety	[55,56]
**Dopaminergic ** **interplay**	Striatal extracellular dopamine ↑ (indirect)	Boosts reward/motivation	May mitigate anergia/anhedonia in older adults with depression	[52]
**Neuroimmune ** **(central)**	5-HT_2A_-linked NF-κB inhibition; microglial modulation (TLR4 ↓, morphology shift)	↓ TNF-α/IL-1β/IL-6; reduced pro-inflammatory transcription	Addresses inflammaging and microglial priming	[57,58]
**Neuroimmune ** **(systemic)**	Acute TNF-α ↓; sustained IL-6/CRP ↓ at ~7 days (dose/time dependent)	Systemic anti-inflammatory signature after single dose	Potentially greater benefit in older adults with elevated baseline inflammation	[59,60]
**Tryptophan ** **metabolism**	5-HT_2A_-linked shift in kynurenine pathway (↓ quinolinic acid/↑ kynurenic acid)	Lowers neurotoxic load; supports neuroprotection	Relevant to late-life depression/AD with kynurenine dysregulation	[9,61]
**Glutamate ** **excitotoxicity**	Modulation of glutamatergic tone	May indirectly reduce excitotoxicity linked to neuroinflammation	Aging brains are more susceptible to excitotoxic injury	[62]

**Table 3:** Adverse effects of psilocybin in older adult patients (≥65 years).

AE Category	Typical Events Reported in Older Adults	Refs.
**Cardiovascular**	Transient increases in blood pressure and heart rate during dosing; occasional SBP >140–180 mmHg	[20,77]
**Gastrointestinal**	Nausea, GI upset; occasional vomiting	[37,77]
**Neurologic/somatic**	Headache (including “next-day headache”), dizziness, fatigue	[31,37]
**Psychological (acute)**	Transient anxiety, emotional discomfort, brief confusion; rare session-limited paranoid/psychotic-like content	[31,48]
**Serious adverse events (SAEs)**	None observed; no HPPD or persistent psychosis	[20,100]
**Drug–drug ** **interactions **	SSRIs/MAOIs/TCAs may blunt or alter acute effects; serotonin toxicity risk theoretical in trials. Psilocin is primarily glucuronidated via UGT1A9/UGT1A10; inducers (e.g., rifampicin) may reduce exposure; inhibitors (e.g., probenecid, diclofenac) may increase exposure	[37,81]

Abbreviations: AE—adverse event; SAE—serious adverse event; GI—gastrointestinal; SBP—systolic blood pressure; HPPD—hallucinogen-persisting perception disorder; SSRI—selective serotonin reuptake inhibitor; MAOI—monoamine oxidase inhibitor; TCA—tricyclic antidepressant; UGT—uridine diphosphate-glucuronosyltransferase.

## Data Availability

The original contributions presented in this study are included in the article. Further inquiries can be directed to the corresponding author.

## Abbreviations

The following abbreviations are used in this manuscript:

AD	Alzheimer’s disease
ALS	amyotrophic lateral sclerosis
BBB	blood–brain barrier
BDNF	brain-derived neurotrophic factor
CRP	C-reactive protein
DMN	default mode network
FDA	Food and Drug Administration
HPA	hypothalamic–pituitary–adrenal
IL-1β	interleukin-1 beta
IL-6	interleukin-6
LPS	lipopolysaccharide
LSD	lysergic acid diethylamide
MCI	mild cognitive impairment
MDD	major depressive disorder
MS	multiple sclerosis
mTOR	mammalian target of rapamycin
NF-κB	nuclear factor-kappa B
NSAIDs	nonsteroidal anti-inflammatory drugs
PD	Parkinson’s disease
SSRI	selective serotonin reuptake inhibitor
TBI	traumatic brain injury
TNF-α	tumor necrosis factor-alpha
TRD	treatment-resistant depression
TrkB	tropomyosin receptor kinase B
WHO	World Health Organization
5-HT	serotonin
5-HT_2A_	serotonin 2A receptor
